# Chondroprotective Effect of Wufu Decoction on Tumor Necrosis Factor‐α‐Induced Chondrocytes *via* the Extracellular Signal Regulated Kinase 1/2 Signaling Pathway

**DOI:** 10.1111/os.12745

**Published:** 2020-07-23

**Authors:** Zheng‐cong Ye, Can‐feng Wang, Lei Han, Guo‐ping Cao, Qin‐rong Shen

**Affiliations:** ^1^ Department of orthopedics Traditional Chinese Medicine Hospital of Xiaoshan District Zhejiang China; ^2^ Department of orthopedics Jiangnan Hospital Affiliated to Zhejiang Chinese Medical University Zhejiang China; ^3^ Department of orthopedics Shaoxing Hospital of Traditional Chinese Medicine Zhejiang China

**Keywords:** Chondrocytes, Collagen II, Osteoarthritis, TNF‐α, Traditional Chinese medicine

## Abstract

**Objective:**

Wufu Decoction (WFD) is a herbal formulation composed of five traditional Chinese herbs that is used clinically for arthritis treatment in China. The current study investigated the chondroprotective effects and the underlying mechanism of WFD for osteoarthritis (OA) therapy.

**Methods:**

The chondroprotective effects of WFD were investigated based on *vitro* study. Following the successful isolation of chondrocytes from rat cartilage tissues and the identification of collagen II expression with immunofluorescence staining, chondrocytes were co‐incubated with tumor necrosis factor‐α (TNF‐α) to induce an inflammation model; WFD was also administered. After the treatment, cell viability was determined by MTT assay, cell apoptosis was assessed by DAPI staining, the concentration of inflammation cytokines interleukin (IL)‐1β and IL‐6 were detected with ELISA assay, the expression of collagen II, MEK1/2‐ERK1/2 signaling pathway proteins was detected using western blotting, and mRNA expression of MMP‐1, MMP‐9 and MMP‐13 were determined with quantitative real‐time polymerase chain reaction.

**Results:**

Wufu Decoction significantly restored the cell viability suppressed by TNF‐α and inhibited the cell apoptosis induced by TNF‐α in chondrocytes. The high concentrations of IL‐1β and IL‐6 in TNF‐α‐induced model cells were significantly decreased in WFD‐treated chondrocytes, and the immunofluorescence staining and western blot results showed that the inhibited expression of collagen II in the TNF‐α‐induced model group was significantly increased in WFD‐treated chondrocytes. The protein expressions of MEK1/2, p‐ERK1/2, and P53 were significantly reduced in the WFD‐treated group compared with those in the model group, and the mRNA expressions of MMP‐1, MMP‐9, and MMP‐13 were also significantly reduced with WFD treatment.

**Conclusion:**

The present study indicated that WFD exerted a chondroprotective effect in TNF‐α‐induced chondrocytes *via* the regulation of the ERK1/2 signaling pathway, suggesting that WFD has therapeutic potential for OA therapy.

## Graphical abstract

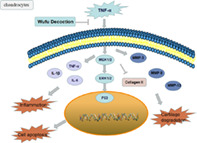

## Introduction

Osteoarthritis (OA) is a common disease of articular joints. It is critically associated with significant morbidity, mortality, and increased healthcare burden in middle aged and elderly people^1^. Joint pain, inflammation, stiffness, deformity, and dysfunction are the main clinical symptoms of OA. Articular cartilage consists of extracellular matrix (ECM) and chondrocytes. Chondrocytes, the only resident cells in the articular cartilage, are important for the structure and function maintenance of articular cartilage through control of ECM synthesis and degradation^2^. The imbalance in synthesis and degradation of articular chondrocytes, ECM, and subchondral bone are responsible for the pathogenesis of OA. Inflammation is a central event in OA, even in the early stages of the disease, with the excessive production of various cytokines. Tumor necrosis factor‐α (TNF‐α) and interleukin (IL)‐1 are known to be elevated in OA and contribute to ECM degradation, matrix metalloproteinase (MMP) synthesis, and collagen II and cartilage loss^3^. Collagen II is the most abundant component of articular cartilage; it is the characteristic protein in chondrocytes and can promote the proliferation and differentiation of chondrocytes. However, high expression of MMP (e.g. MMP‐3, MMP‐9, and MMP‐13) in chondrocytes can lead to the degradation of collagen II and ECM^4^. Studies have also found that the extracellular signal regulated kinase (ERK)1/2 signaling pathway is activated in OA. The phosphorylation of the ERK1/2 signaling pathway is the mediator in the production of abnormal levels of MMP^5^. The ERK1/2 signaling pathway is also found to promote the proliferation and apoptosis of chondrocytes, thus involving the pathogenesis of OA.

The American College of Rheumatology and the European League Against Rheumatism suggest non‐steroidal anti‐inflammatory drugs (NSAID) T^6^. Prior to the use of Western medicine in China for OA treatment, there was a long history of application of traditional Chinese medicine in clinical therapy for OA. Wufu Decoction (WFD), composed of five traditional Chinese herbs, *Codonopsis pilosula*, *Rehmanniae radix*, *Atractylodes macrocephaia*, *Angelica sinensis* and *Radix Glycyrrhizae*, is a classical formula originating from the time of the Ming dynasty of China. Our previous studies demonstrated that WFD has a therapeutic effect on OA, but the precise mechanism underlying the effects of WFD on inflammation‐induced OA remains to be elucidated ^7^, ^8^. Therefore, in this study, we hypothesize that: (i) the chondrocytes isolated from rat cartilage tissues co‐incubated with TNF‐α could induce an articular chondrocytes degradation and inflammation model; (ii) WFD has a chondroprotective effect against OA in TNF‐α‐induced rat articular chondrocytes; and (iii) the underlying molecular mechanisms of WFD's chondroprotective effects might be related to the regulation of the ERK1/2 signaling pathway. This study provides a reference for the clinical application of WFD in OA therapy.

## Materials and Methods

### *Preparation of Wufu Decoction‐Containing Serum*

Following 1 week of acclimation, 30 Wistar rats (weighing 150–180 g) were randomly divided into three groups (n = 10): a blank group, a positive control group, and an experiment group. The rats in these three groups were intragastrically injected with saline, D‐Glucosamine sulfate (DGS, 1.5 g/[kg·d]), and WFD (2.64 g/[kg·d]), respectively. All rats were treated for 7 consecutive days, and blood samples were collected 2 h after the last administration. Drug‐containing serum was obtained from the centrifuged blood samples of these three groups of rats, and was stored at −80°C for subsequent *in vitro* experiments.

### *Chondrocytes Isolation and Identification*

The bilateral knee joint cartilage was obtained from the joints of rats after they were killed. Then the cartilage was washed with phosphate buffered saline (PBS) and minced into 1‐mm^3^ fragments. The supernatants were removed and the reserved cartilage tissues were submitted to digestion with collagenase II at 37°C for 4 h. After that, the suspension liquid from digestion cells was filtrated, and centrifuged at 56 xg for 5 min to remove the supernatant. Then the chondrocytes were obtained and cultured at a density of 1 × 10^5^ cells/dish in the DMEM medium (Hyclone) containing 10% fetal bovine serum, 100 μg/mL streptomycin, and 100 U/mL penicillin in a 37°C, 5% CO_2_ incubator. Afterwards, the culture medium was replaced every 2–3 days. The chondrocyte morphology was observed under an AE2000 light microscope (Motic); the primary chondrocytes were termed passage 1 (P1), and P2 chondrocytes were used for the subsequent experiments.

### *Grouping and Treatments*

Chondrocytes were divided into four groups: a control group, which was administered 10% of the serum (100 μL/mL) from the blank group; a model group, administered 20 μg/L TNF‐α and 10% of the serum (100 μL/mL) from the blank group; a DGS group, administered 20 μg/L TNF‐α and 10% of the serum (100 μL/mL) from the positive group; and a WFD group, administrated 20 μg/L TNF‐α and 10% of the serum (100 μL/mL) from the experiment group. All group cells were treated with drug‐containing serum for 48 h.

### *Immunofluorescence Staining*

The differentiation status of chondrocytes was further determined by examining the expression of type II collagen by immunofluorescence assay. Chondrocytes were treated with or without TNF‐α for 48 h, and then incubated with antibodies against collagen II. Then 1 μg/mL DAPI was added to each well for cell nucleus staining. The staining reagent was removed after 2 min and the well was washed with PBS. Afterwards, an anti‐fluorescence quenching seal tablet was added to the glass coverslips, and fluorescence images were recorded with an Axio Observer A1 fluorescence microscope (ZEISS).

### *MTT Assay*

The cell viability of each group of chondrocytes was determined by MTT assay. The chondrocytes were incubated with the WFD or DGS for 12 h, 24 h, 48 h, and 72 h, respectively. The wells were emptied and 100 μL 0.5% MTT solution was then added before continuing to incubate for 4 h. The optical density (OD) value was measured at 490 nm using a CMaxPius ELISA reader (SpectiaMax, USA).

### *ELISA Assay*

After the 48 h of incubation, the chondrocytes were centrifuged at 1500 *g* for 10 min to obtain the supernatants. Concentrations of IL‐1β and IL‐6 (MEIMIAN, China) were detected with the commercial kits in accordance with the relevant manufacturer's instructions.

### *Cell Apoptosis Assay*

The chondrocytes in each group were seeded into six‐well plates at a density of 1.2 × 10^6^. After 48 h of incubation, the medium was removed and the cells were fixed in 4% paraformaldehyde for 30 min and washed with PBS three times. Subsequently, the chondrocytes were stained with 1 μg/mL DAPI (SIGMA, USA) for 2 min and then washed with PBS. The cellular morphology was observed under an inverted fluorescent microscope.

### *Western Blotting*

The chondrocytes were washed with PBS and lysed to obtain the total proteins, and the protein concentration was determined with a bicinchoninic acid assay. Then protein samples were separated by 5% SDS‐polyacrylamide gel electrophoresis (PAGE) through electrophoresis at 80 V and transferred to a polyvinylidene fluoride (PVDF) membrane. After the membranes were blocked with 5% non‐fat milk for 2 h, the primary antibodies against collagen II, MEK1/2, ERK1/2, p‐ERK1/2 and P53 (CST signaling), were incubated with the membrane at 4°C overnight. TBST was used to wash the membranes three times. After that, the secondary antibodies were added and further incubated for 1 h at room temperature. The membranes were rinsed three times with TBST and the reacted protein bands were further visualized using an enhanced chemiluminescence western blot substrate. The brands were semi‐quantified with Image J software and normalized to the β‐actin.

### *Quantitative Polymerase Chain Reaction Analysis*

TRIzol reagent (1 μg) was used to extract the total RNA of chondrocytes in the four groups incubated for 48 h according to the manufacturer's instructions. Total RNA (1 μg) was used for cDNA synthesis by the Reverse Transcription System ABI7500 (Roche, Switzerland) in accordance with the experimental procedures for quantitative real‐time polymerase chain reaction (PCR) analysis. The primer sequences used were as follows: MMP‐3, forward 5′‐ATGCAGGGAAAGTGACCCAC‐3′, reverse 5′‐CGACGCCCTCCATGAAAAGA‐3′; MMP‐9, forward 5′‐GTGCCCTGGAACTCACACAAC‐3′, reverse 5′‐CCAGAAGTATTTGTCATGGCAGAA‐3′; MMP‐13, forward 5′‐TGCTGCATACGAGCATCCAT‐3′, reverse 5′‐TGTCCTCAAAGTGAACCGCA‐3′; β‐actin, forward 5′‐GGGAAATCGTGCGTGACATT‐3′, reverse 5′‐GCGGCAGTGGCCATCTC‐3′. mRNA expression fold changes were calculated using the 2^–ΔΔCt^ method after data was normalized to the average of the reference gene β‐actin.

### *Statistical Analysis*

All data are shown as mean ± standard derivation (SD). Statistical analyses were performed using SPSS 19.0 software. The Student *t*‐test was used to compare the differences between the two groups. A *P*‐value less than 0.05 was considered statistically significant.

## Results

### *Identification of Chondrocytes*

**Fig 1:**
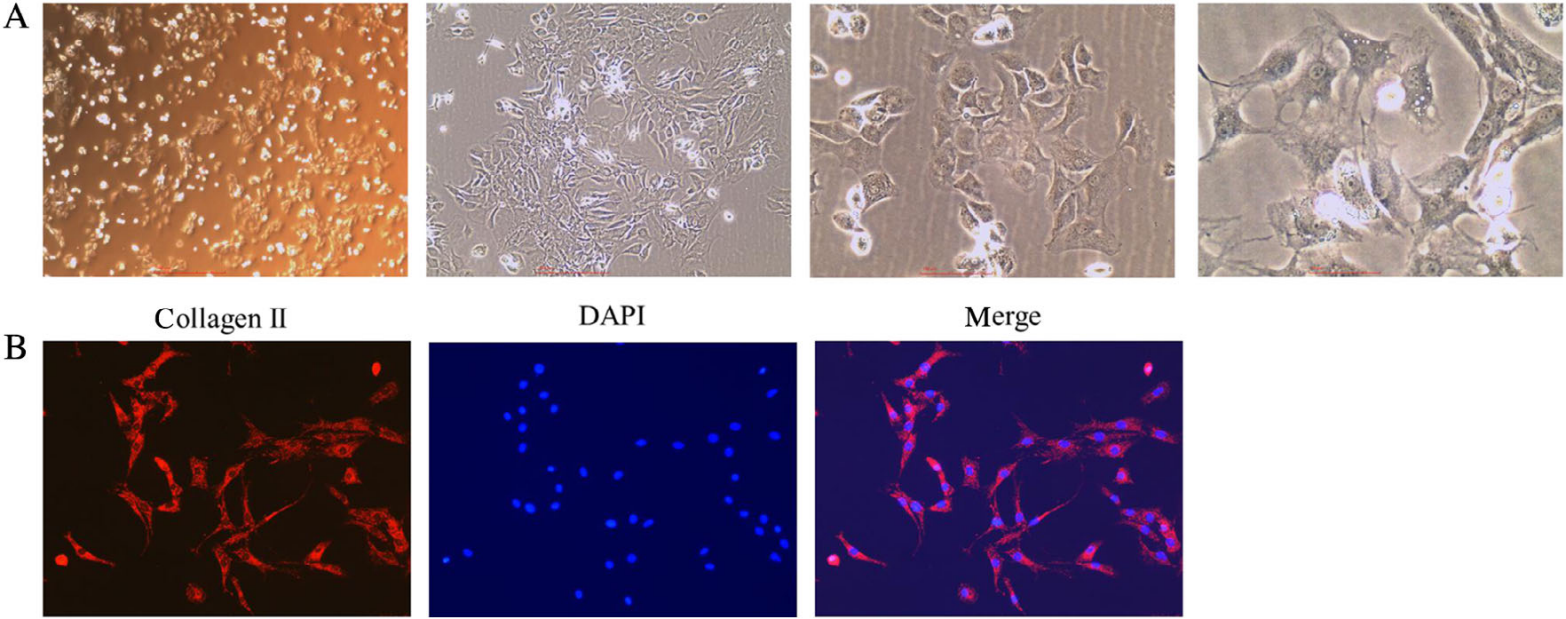
The morphology and identification of tumor necrosis factor‐α (TNF‐α)‐treated chondrocytes. (A) Morphology of primary cultured chondrocytes (magnification, ×40, 100, 200, 400, respectively). (B) Immunofluorescence assay of collagen II in chondrocytes.

The morphology of the primary chondrocytes was observed with a light microscope under different magnifications (×40, 100, 200, and 400). The primary cultured chondrocytes exhibited a normal status, with polygon, irregular or paving stone‐like shapes, large and round cell nuclei, copious cytoplasm, and multiple nucleoli (Fig. 1A). Immunofluorescence staining also showed that the cell nuclei were stained with blue fluorescence, and collagen II was extensively expressed in cell cytoplasm (Fig. 1B).

### *Effect of Wufu Decoction on Cell Viability in TNF‐α‐Induced Chondrocytes*

**Fig 2:**
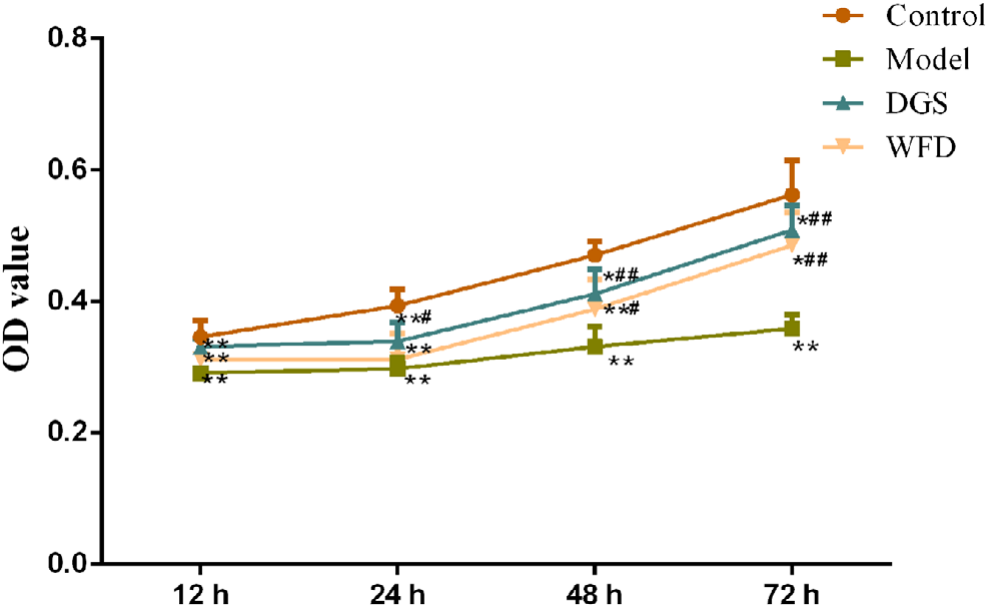
Wufu Decoction (WFD) enhanced the cell viability of tumor necrosis factor‐α (TNF‐α)‐treated chondrocytes. MTT assay was performed to detect the optical density (OD) value of the chondrocytes. Comparing with the control group, *P < 0.05, **P < 0.01; comparing with the model group, ^#^ *P* < 0.05, ^##^ *P* < 0.01.

The viability of chondrocytes was detected with MTT assay (Fig. 2). It showed that TNF‐α being added to the chondrocyte culture medium induced a significant inhibition of cell viability in the model group compared with the control group for 12 h, 24 h, 48 h, and 72 h incubation, and the OD value in the control group was 1.21 times, 1.33 times, 1.41 times, and 1.58 times that of model group, respectively (*P* < 0.01). After 48 h treatment, the OD values in the WFD group were increased significantly compared with the model group, and the OD value in the WFD group was 1.18 times that of the model group (*P* < 0.05); after 72 h treatment, the OD value in the WFD group was also increased significantly compared with the model group, and the OD value in the WFD group was 1.36 times that of model group (*P* < 0.01).

### *Wufu Decoction Inhibited the IL‐1β, IL‐6 Levels in Tumor Necrosis Factor‐α‐Induced Chondrocytes*

**Fig 3:**
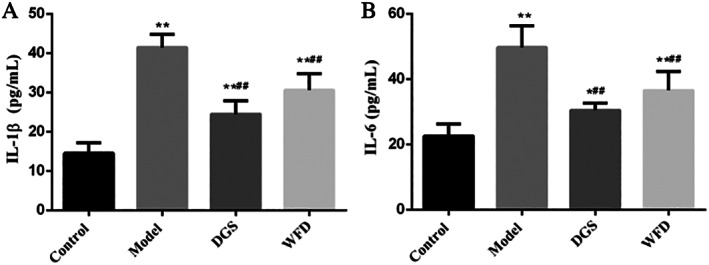
Effect of Wufu Decoction (WFD) on interleukin (IL)‐1β (A) and IL‐6 (B) expression in tumor necrosis factor‐α (TNF‐α)‐treated chondrocytes. Comparing with the control group, *P < 0.05, **P < 0.01; comparing with the model group, ^#^ *P* < 0.05, ^##^ *P* < 0.01.

The effect of WFD on the inflammation in TNF‐α‐induced chondrocytes was also evaluated with detection of the levels of IL‐1β and IL‐6 (Fig. 3). After the induction of TNF‐α in chondrocytes, the level of IL‐1β was increased significantly in the model group compared with the control group, and the average level of IL‐1β in the model group was 2.85 times that of the control group (41.42 ± 3.33 pg/mL *vs* 14.54 ± 2.63 pg/mL, *P* < 0.01; Fig. 3A). However, following the treatment with WFD, the elevated IL‐1β level was decreased significantly compared to the model group; the average level of IL‐1β in the WFD group was 0.74 times that of the model group (30.62 ± 4.15 pg/mL *vs* 41.42 ± 3.33 pg/mL, *P* < 0.01). A similar tendency was observed for the concentrations of IL‐6 among these groups (Fig. 3B).

### *Wufu Decoction Inhibited the Apoptosis of TNF‐α‐Induced Chondrocytes*

**Fig 4:**
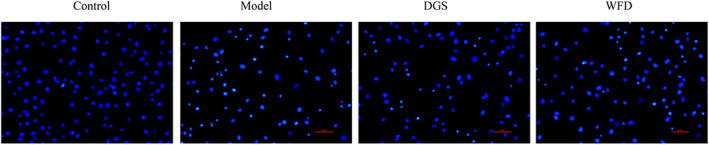
Wufu Decoction (WFD) inhibited the apoptosis of tumor necrosis factor‐α (TNF‐α)‐induced chondrocytes. DAPI staining was used to detect the apoptosis of the cells (magnification, x200).

To further evaluate the effect of WFD on the apoptosis of chondrocytes, DAPI staining assay was performed after the 48 h treatment of chondrocytes. The results showed that compared to the chondrocytes in the control group, the model group cells exhibited typical alterations, including reduced number of bright blue stained cell nuclei and cellular volume, and condensed or fragmented cell nuclei, but this phenomenon was attenuated with the administration of WFD and DGS (Fig. 4).

### *Effect of Wufu Decoction on the Expression of Collagen II in TNF‐α‐Induced Chondrocytes*

**Fig 5:**
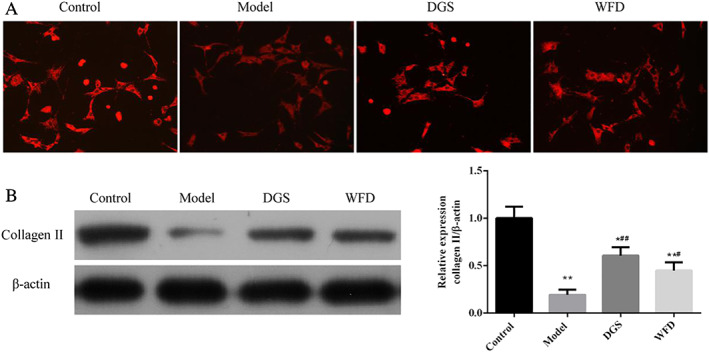
Wufu Decoction (WFD) inhibited the expression of collagen II in chondrocytes. (A) Immunofluorescence analysis of the expression of collagen II. (B) Protein expression of collagen II. Comparing with control group, *P < 0.05, **P < 0.01; comparing with model group, ^#^ *P* < 0.05, ^##^ *P* < 0.01.

Immunofluorescence assay showed that collagen II was located in cell cytoplasm, and the immunofluorescence intensity of collagen II was decreased in the model group compared with the control group, but the intensity was increased with the WFD and DGS treatment compared with the model group (Fig. 5A). The protein expression of collagen II in the control group was nearly five times that of the model group with TNF‐α co‐incubation (1.00 ± 0.12 *vs* 0.19 ± 0.05, *P* < 0.01; Fig. 5B). However, the administration of WFD in TNF‐α‐induced chondrocytes significantly increased the expression of collagen II compared to the model group, and the expression ratio was 2.34 times that of the model group (0.45 ± 0.08 *vs* 0.19 ± 0.05, *P* < 0.05).

### *Effect of Wufu Decoction on the Protein Expression of MEK1/2, ERK1/2, p‐ERK1/2, and P53 in TNF‐α‐Induced Chondrocytes*

**Fig 6:**
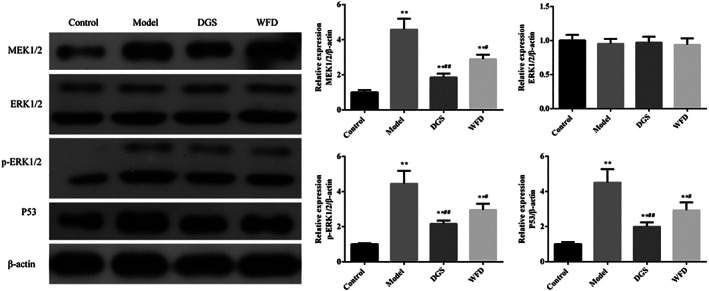
Effect of Wufu Decoction (WFD) on the protein expression of MEK1/2, ERK1/2, p‐ERK1/2, and P53 in tumor necrosis factor‐α (TNF‐α)‐induced chondrocytes. Comparing with the control group, *P < 0.05, **P < 0.01; comparing with the model group, ^#^ *P* < 0.05, ^##^ *P* < 0.01.

Western blot analysis was performed to evaluate the effect of WFD on the ERK1/2 signaling pathway (Fig. 6). It showed that the protein expression of MEK1/2 (4.58 ± 0.62 *vs* 1.00 ± 0.13, 4.58 times), p‐ERK1/2 (4.44 ± 0.73 *vs* 1.00 ± 0.05, 4.44 times), and P53 (4.51 ± 0.75 *vs* 1.00 ± 0.11, 4.51 times) were significantly increased in the model group chondrocytes compared to those in the untreated control group (*P* < 0.01). In the WFD group, MEK1/2, p‐ERK1/2, and P53 displayed lower protein expression levels compared with the model group, and the expression ratio was 0.63 times (2.89 ± 0.25 *vs* 4.58 ± 0.62, *P* < 0.05), 0.66 times (2.95 ± 0.36 *vs* 4.44 ± 0.73, *P* < 0.05), and 0.65 times (2.93 ± 0.44 *vs* 4.51 ± 0.75, *P* < 0.05) that of the model group, respectively. In addition, significantly decreased expression of MEK1/2, p‐ERK1/2, and P53 were also observed in the DGS group compared with the model group (*P* < 0.01).

### *Wufu Decoction Inhibited the mRNA Expression of MMP‐3, MMP‐9, and MMP‐13 in TNF‐α‐Induced Chondrocytes*

**Fig 7:**
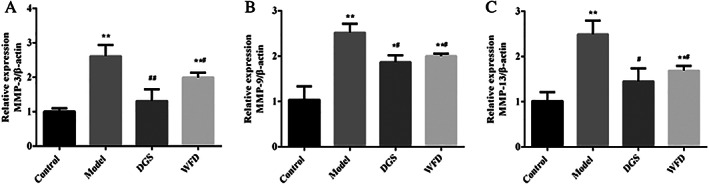
Effect of Wufu Decoction (WFD) on the expression of the mRNA expression of MMP‐3, MMP‐9, and MMP‐13 in tumor necrosis factor‐α (TNF‐α)‐induced chondrocytes. Comparing with the control group, *P < 0.05, **P < 0.01; comparing with the model group, ^#^ *P* < 0.05, ^##^ *P* < 0.01.

The effect of WFD on MMP‐3, MMP‐9, and MMP‐13 mRNA expression were determined by quantitative PCR analysis (Fig. 7). TNF‐α significantly increased the mRNA expression of MMP‐3 (2.61 ± 0.33 *vs* 1.00 ± 0.09, 2.61 times), MMP‐9 (2.52 ± 0.19 *vs* 1.03 ± 0.30, 2.44 times), and MMP‐13 (2.49 ± 0.30 *vs* 1.01 ± 0.20, 2.46 times) in the model group compared to the untreated control group (*P* < 0.01). However, the administration of WFD inhibited the MMP‐3, MMP‐9, and MMP‐13 mRNA expression levels. In the WFD group, the decreased mRNA expression of MMP‐3 was 0.76 times that in the model group (1.99 ± 0.13 *vs* 2.61 ± 0.33, *P* < 0.05), MMP‐9 was 0.74 times that in the model group (2.00 ± 0.05 *vs* 2.52 ± 0.19, *P* < 0.05), and MMP‐13 was 0.67 times that in the model group (1.69 ± 0.01 *vs* 2.49 ± 0.30, *P* < 0.05), respectively.

## Discussion

Currently, the management of OA patients mainly focuses on the combination of pharmacologic therapy, non‐pharmacologic therapy, and surgery, with the goal of preventing disease and slowing down the progress of the OA^6^, ^9^, ^10^. Compared with non‐pharmacologic treatment, the establishing guidelines for pharmacologic therapy remain challenging due to the efficacy and safety of pharmacologic agents in the management of OA^11^. Therefore, exploring potential drugs that are effective and safe for OA patients' therapy and rehabilitation are the future directions of OA research. In the present study, the protective effect of WFD, a formula composed of five traditional Chinese herbs, was explored for OA treatment in *in vitro* experiments. WFD administration effectively attenuated the inflammation, improved the cell viability, and inhibited the cell apoptosis and the expression of collagen II and MMP in chondrocytes treated with TNF‐α.

Wufu Decoction is composed of five traditional Chinese herbs, *Codonopsis pilosula*, *Radix Rehmanniae*, *Atractylodes macrocephaia*, *Angelica sinensis*, and *Radix Glycyrrhizae*, and has abundant pharmacological activities. Some previous studies also implied the chondroprotective and anti‐inflammatory activities of these herbs. Jhun *et al* found that *Radix Rehmanniae* and *Notoginseng Radix* extracts significantly lessened the damage to articular cartilage (demonstrated visually and histopathologically), suppressed the expression of MMP‐3, IL‐1β, and IL‐6 in OA cartilage, and upregulated the tissue inhibitor of metalloproteinase (TIMP) 1 and TIMP‐3 levels in IL‐1β‐stimulated human OA chondrocytes^12^. *Atractylodes macrocephaia* was found: to recover the elevated levels of hyaluronic acid, laminin, type IV collagen, type III procollagen, and TGF‐β1; to suppress procollagen I, collagen III, and TIMP‐1 expression; and to improve the TIMP‐1/MMP‐13 ratio in rat liver fibrosis^13^. *Angelica sinensis* polysaccharide can protect chondrocytes from H_2_O_2_‐induced oxidative stress injury through its anti‐oxidant, anti‐apoptotic, and anti‐inflammatory effects in vitro^14^, ^15^. Another complex herbal decoction containing *Angelica sinensis* was proved to have chondroprotective effects through enhancing the growth of SW1353 chondrocytes, significantly inhibiting the IL‐1β‐induced MMP‐1 expression, and anti‐inflammatory effects through reducing the activation of inflammatory mediators such as NF‐kB, IL‐1β, IL‐6, PGE2, and NO in LPS‐induced RAW264.7 cells^16^. These previous studies and our present study have indicated that WFD, composed of five herbs, has potential chondroprotective effects in OA, through anti‐apoptosis, anti‐inflammation, and the regulation of cartilage degradation.

Inflammation has a significant role in the pathogenesis of OA, and the pro‐inflammatory cytokine TNF‐α is commonly used in culture models to mimic the circumstances leading to *in vivo* cartilage degradation^17^. Therefore, this study was performed based on the culture of rat chondrocytes with the presence of TNF‐α. As confirmed by our study, TNF‐α significantly inhibited the viability of chondrocytes, the induction of cell apoptosis, the inflammation response, and the abnormal expression of collagen II and MMP in chondrocytes, which were proved to destruct the articular cartilage during the progression of OA. Elevated levels of TNF‐α, IL‐1, and IL‐6 have been found in the synovial fluid, synovial membrane, subchondral bone, and cartilage of OA patients, confirming their important roles in OA pathogenesis. TNF‐α in synovial fluid was found to be associated with increased risk for subsequent osteophyte progression and reduced activity in daily living in OA meniscectomized patients^18^. Higher serum levels of TNF‐α were also observed in knee OA patients than in healthy controls; TNF‐α levels showed 74.1% sensitivity and 76.0% specificity in a receiver operating characteristic curve analysis and could serve as an independent predictor of severe knee OA^19^. Therefore, the TNF‐α inhibitors or agents, with the ability to competitively bind to TNF‐α receptors (TNFR1 and TNFR2) and suppress the pro‐inflammatory function of TNF‐α, could provide new insight into the pathogenesis and therapeutic strategy of OA^20^, ^21^. Except for the induction of other cytokines, TNF‐α is also responsible for the production of MMP and inhibiting the synthesis of collagen II and SOX9, thus is critically important in cartilage matrix degradation. Furthermore, TNF‐α can act on the cell surface to activate MAPK signaling pathways and regulate the expression of downstream targets such as c‐jun, JNK, ERK, and p38^22^. Studies showed that the activation of the ERK1/2 signaling pathway promotes MMP production and inflammatory factor expression, thus contributing to the cartilage destruction during OA^5^, ^23^.

The pathological destruction of joint tissues in OA is in large part due to the degradation of ECM components by elevated MMP. MMP were initially characterized as matrix‐degrading proteinases, but recent studies have shown that their actions on ECM could release growth factors and activate inflammatory cytokines and chemokines^24^, ^25^. In cartilage of OA, elevated levels of MMP, including MMP‐1, MMP‐3, MMP‐9, and MMP‐13, could be observed. TIMP, as the name implies, are a group of inhibitors that inhibit the MMP activity specifically. The MMP and TIMP activity is in steady balanced state in non‐arthritic human cartilage. However, in OA cartilage, this balance is broken and shifts towards MMP, causing degradation of ECM. Research exploring inhibitors targeting MMP as therapeutic agents in arthritic diseases served a new strategy in OA treatment; however, the outcome transferring into early clinical trials was frustrating^26^. Therefore, more comprehensive knowledge of these MMP in chondrocytes and their roles in the OA process is still necessary.

In conclusion, in the present study, we investigated the chondroprotective effects of WFD against OA in TNF‐α‐induced rat articular chondrocytes, and the results showed that WFD significantly attenuated inflammation and cartilage injury by inhibiting the elevated expression of IL‐1β, IL‐6, collagen II, MMP‐1, MMP‐9, MMP‐13, the apoptosis of chondrocytes, attenuating cell viability, and regulating of MEK1/2‐ERK1/2 signaling pathway in TNF‐α treated chondrocytes. This study revealed that WFD could alleviate OA injury *via* the ERK1/2 signaling pathway, but further in‐depth studies are required to investigate the precise roles of WFD in OA therapy.
